# Non-Hodgkin's lymphoma presenting as a primary bladder tumor: a case report

**DOI:** 10.1186/1752-1947-4-114

**Published:** 2010-04-26

**Authors:** José A Díaz-Peromingo, Javier Tato-Rodríguez, Paula M Pesqueira-Fontán, Sonia Molinos-Castro, María C Gayol-Fernández, Juliusz P Struzik

**Affiliations:** 1 Department of Internal Medicine, Hospital da Barbanza, Oleiros, Riveira, 15993, Spain; 2 Department of Urology, Hospital da Barbanza, Oleiros, Riveira, 15993, Spain

## Abstract

**Introduction:**

Primary lymphoma of the bladder represents 0.2% of all bladder malignancies. Secondary involvement of the bladder by malignant lymphoma occurs in 10% to 50% of cases. Most lymphomas of the bladder are non-Hodgkin's lymphomas of the B-cell type, with preponderance among women. The impact of positron emission tomography (PET) on tumor staging has recently become very important due to its use in the study of diagnosis extension and individual therapy design.

**Case presentation:**

We report the case of a 79-year-old Caucasian man with intermittent haematuria as the presenting symptom of non-Hodgkin's lymphoma of the bladder. He was first diagnosed with primary lymphoma of the bladder using the current staging method, but a positron emission tomography study subsequently revealed that he instead had a secondary involvement of the bladder.

**Conclusion:**

The staging of non-Hodgkin's lymphomas, which is useful in order to plan accurate therapy, has been changing since the introduction of positron emission tomography scanning. Primary lymphomas of the bladder, although very rare, may be even more uncommon when this imaging technique is used to assess the extension of the disease. Although the interpretation of this technique has some limitations that should be taken into account, the extensive use of positron emission tomography should nonetheless help improve the diagnosis of this disease.

## Introduction

Most tumors of the bladder are derived from the epithelium. Non-epithelial tumors are extremely rare. Among these, leiomyosarcomas are the most common in adults and rhabdomyosarcomas are the most common in children [1]. Metastatic tumors more frequently affect the bladder neck and the deep trigone and represent approximately 15% of all known bladder malignancies [2].

Meanwhile, primary lymphomas of the bladder are very rare and were first described by Eve in 1885 [3]. They represent 0.2% of all known bladder cancers [4]. Secondary bladder involvement is reported in 10% to 50% of cases, with a maximum incidence between the fourth and sixth decades of life [5,6]. Most lymphomas of the bladder are low-grade non-Hodgkin's lymphomas (NHL) of the B-cell type [1,7]. Women are affected more frequently than men [4,8].

The most frequent symptoms of bladder lymphomas are gross haematuria followed by concomitant urinary tract infection, dysuria and increased urinary frequency [9]. Other complications like hydronephrosis, fistulas or involvement of the entire bladder are very rare [10].

Usually, the diagnosis of primary lymphoma of the bladder is one of exclusion. It is made in the absence of any other nodal or extranodal involvement after biopsy with immunohistochemical study, and after a negative study of disease extension, which includes bone marrow biopsy and computed tomography (CT) [11]. This approach is changing with the introduction of positron emission tomography (PET) to assess for other nodal or extranodal involvement when a possible primary lymphoma of the bladder is suspected. In this case report, we present a case of primary lymphoma of the bladder in which PET scanning changed the diagnosis to extended NHL. We review the literature focusing on the use of PET in the assessment of tumor extension.

## Case presentation

A 79-year-old Caucasian, Spanish man was admitted to our hospital because of intermittent painful gross haematuria lasting for seven days. He smoked 20 cigarettes per day and reported having an appendectomy 40 years prior to presentation. He had worked as a seaman in his youth and was currently retired. He took no medications.

Results of his general examination were normal. His rectal examination revealed an enlarged prostate, grade I/IV, with no masses or nodules. Results of his analytical studies including serum chemistry, prostate specific antigen (PSA), and coagulation studies were normal. A mild anaemia (hemoglobin = 11.3 g/L) with a normal mean corpuscular volume was reported. His erythrocyte sedimentation rate (ESR) was 67 mm per hour. An ultrasonographic study of his abdomen had normal results, except for an irregular border of the right lateral wall of his bladder.

Meanwhile, his intermittent gross haematuria continued, sometimes with associated blood clots. A cystoscopy procedure was performed and an irregular mass affecting his right antero-lateral wall was found. Biopsy revealed a diffuse, large B-cell lymphoma with the following immunohistochemical findings: CD20-, BCL6-, and DC10-positive. Meanwhile, his MIB1 had a high proliferation level. CT scans of his chest, abdomen and pelvis were performed and showed neither enlarged nodes nor liver or spleen involvement. No metastatic lesions were likewise found. His peripheral blood smear test was normal, as were his direct and indirect Coombs tests, serum protein counts, and plasma serum immunoglobulins. His β2-microglobulin was 2.79 mg/dL (normal range = <0.27 mg/dL).

**Figure 1:**
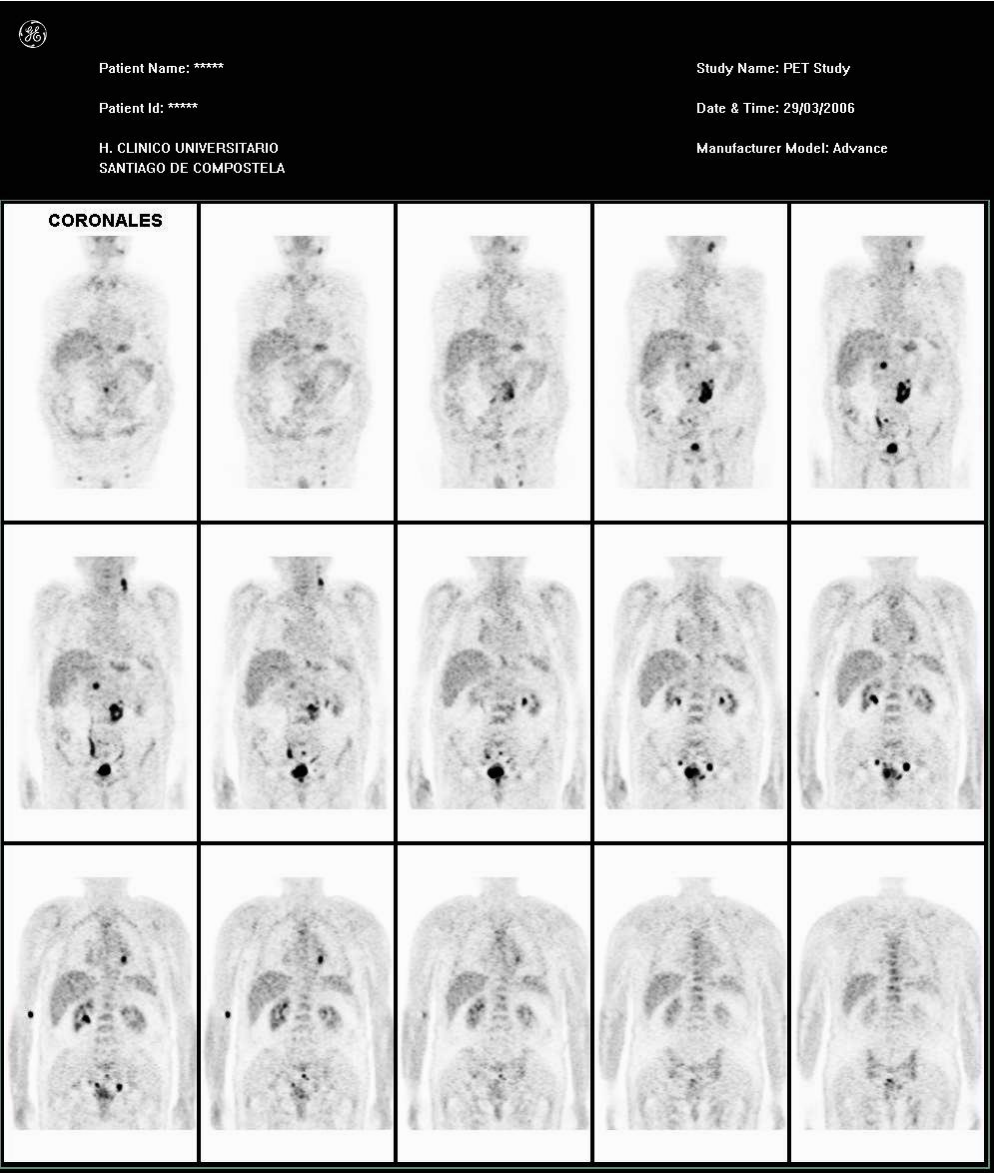
**Coronal view shows multiple areas of increased metabolism affecting the neck, mediastinum, left suprarenal gland, abdominal lymph nodes and the bladder**.

**Figure 2:**
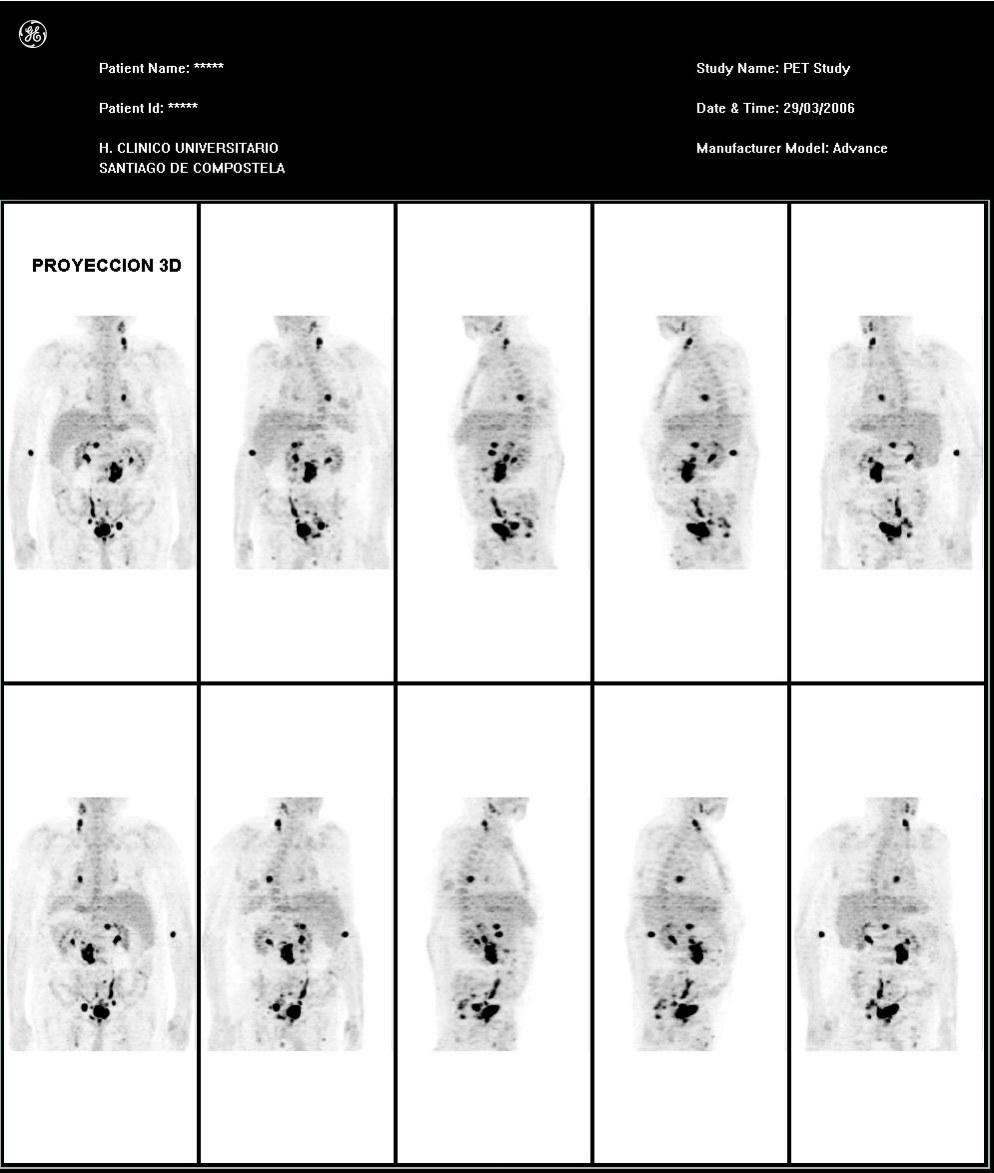
**This image shows the PET findings in a three-dimensional projection view**.

Results of a whole body PET study of our patient (Figures 1 and 2) revealed two nodes with increased metabolism in the left part of his neck, and another area close to his left supraclavicular space, which was suggestive of nodal involvement. An enlarged left mediastinal lymph node was also found on our patient. His left suprarenal gland showed hypermetabolism. His abdomen appeared to have multiple lymph node infiltrates affecting his lumbar region in particular, both his iliac lymphatic chains, and those close to his bladder with associated hypermetabolism of his bladder walls. Results of his bone marrow biopsy were also normal.

Our patient was started on a treatment with CNOP (cyclophosphamide, mitoxantrone, vincristine and prednisolone) and monoclonal antibodies anti-CD20. He showed good tolerance and initial response to this treatment.

## Conclusion

Our patient showed no abnormalities on the CT study. Nevertheless, significant nodal and extranodal (suprarenal gland) extension of his disease was discovered upon performing a PET study.

PET with 2- [fluorine 18] fluoro-2-deoxy-D-glucose (FDG) is increasingly being used in combination with CT to evaluate thoracic and abdominopelvic malignancies [12]. A common systemic malignancy involving the pelvis is NHL. Whole-body PET is useful in the detection of a wide variety of both primary and metastatic malignancies because of the high glycolytic rate that the malignant tissue presents. The presence of FDG uptake in benign inflammatory conditions may limit the specificity of PET. Sensitivity for the detection of malignant lesions is about 97% and the positive predictive value is 94%. This technique is promising both in determining the nature of a localized lesion, as well as in defining the systemic extent of a malignant disease [13].

A number of studies have shown FDG-PET to be useful and in fact superior to CT for primary staging and assessment of disease extension in both Hodgkin's disease and NHL. The technique is reported to have sensitivities of 82% to 99% and specificities of 99% to 100% [14]. Although data regarding the use of in-line FDG-PET-CT systems in evaluating lymphoma are inconclusive, preliminary results appear to indicate that this technique is useful when a guided biopsy procedure is needed [15]. In one study that compared the diagnostic performance of PET alone, CT alone, and fused images for restaging or follow-up of patients with malignant lymphoma, 50 patients with NHL were included. In this study, the interpretation of PET alone (sensitivity = 86.1%, specificity = 99.4%, accuracy = 91.0%), and fused images (98.0%, 99.4%, and 99.2%, respectively) yielded significantly better diagnostic performance than CT alone (59.4%, 96.1%, 91.0%; P < 0.001). In particular, findings in cervical, supraclavicular and extranodal regions were more accurately identified using PET (P < 0.05) [16]. With regard to staging, FDG-PET is more sensitive and specific than conventional staging methods in FDG avid lymphomas such as Hodgkin's lymphoma and most aggressive NHLs.

In assessing a patient's response to therapy, FDG-PET at the end of treatment seems to aid considerably in differentiating between residual masses with and without residual lymphoma. Concerning treatment planning, meanwhile, in the context of a combined-modality therapy, radiotherapy for lymphomas is moving towards more conformal techniques to reduce the irradiated volume and to include only the macroscopic lymphoma. In this context, accurate imaging is essential, and FDG-PET in combination with CT scan is increasingly being used. The availability of PET and CT scanners suited for virtual simulation has aided in this process [17].

The limitations of FDG-PET in detecting lymphomas have included variable FDG uptake in low-grade lymphomas; physiologic activity in muscles, bone marrow, bowels, and the urinary system; and FDG uptake in inflammatory or infectious processes, any of which may mask or mimic tumor signals [18]. Another limitation in the analysis of the pelvis and the urinary tract is the physiological excretion of radiotracers [19].

This case suggests the need for extensive lymphoma staging, and especially the need for PET implementation, in order to make an accurate diagnosis of the extension of the disease and to properly design a course of treatment.

## Competing interests

The authors declare that they have no competing interests.

## Authors' contributions

JADP, JTR and PPF analyzed patient data on aematological disease and PET interpretation. SMC, MCGF and JPS reviewed the literature related to the clinical case. JADP, JTR and PPF were major contributors in writing the manuscript. JPS provided help in translating the manuscript into English. All authors read and approved the final manuscript.

## Consent

Written informed consent was obtained from our patient for publication of this case report and any accompanying images. A copy of the written consent is available for review by the Editor-in-Chief of this journal.

## References

[B1] Mourad WA Khalil S Radwi A Peracha A Ezzat A Primary T-cell lymphoma of the urinary bladder Am J Surg Pathol 1998 22 373 377 10.1097/00000478-199803000-00014 9500781

[B2] Bates AW Baithun SI Secondary neoplasms of the bladder are histological mimics of non-transitional cell primary tumors: clinicopathological and histological features of 282 cases Histopathol 2000 36 32 40 10.1046/j.1365-2559.2000.00797.x 10632749

[B3] Jacobs A Symington T Primary lymphosarcoma of urinary bladder Br J Urol 1953 25 119 126 10.1111/j.1464-410X.1953.tb09253.x 13059372

[B4] Kuhara H Tamura Z Suchi T Hattori R Kinukawa T Primary malignant lymphoma of the urinary bladder: a case report Acta Pathol Jpn 1990 40 764 769 2291406 10.1111/j.1440-1827.1990.tb01541.x

[B5] Aigen AB Phillips M Primary malignant lymphoma of urinary bladder Urol 1986 28 235 237 10.1016/0090-4295(86)90050-6 3750605

[B6] Downs TM Kibel AS De Wolf WC Primary lymphoma of the bladder: a unique cystoscopic appearance Urol 1997 49 276 278 10.1016/S0090-4295(96)00449-9 9037297

[B7] Fernández Aceñero MJ Martín Rodilla C López García-Asenjo J Coca Menchero S Sanz Esponera J Primary malignant lymphoma of the bladder: report of 3 cases Pathol Res Pract 1996 192 160 163 8692717 10.1016/S0344-0338(96)80211-1

[B8] Freeman C Berg JW Cutler SJ Occurrence and prognosis of extranodal lymphomas Cancer 1972 29 252 260 10.1002/1097-0142(197201)29:1<252::AID-CNCR2820290138>3.0.CO;2-# 5007387

[B9] Santino AM Shumaker EJ Garces J Primary malignant lymphoma of the bladder J Urol 1970 103 310 313 5412748 10.1016/s0022-5347(17)61949-9

[B10] Arda K Ozdemir G Güneş Z Ozdemir H Primary malignant lymphoma of the bladder: a case report and review of the literature Int Urol Nephrol 1997 29 319 322 10.1007/BF02550929 9285304

[B11] Evans DA Moore AT The first case of vesicovaginal fistula in a patient with primary lymphoma of the bladder: a case report J Med Case Reports 2007 27 1 105 10.1186/1752-1947-1-105 PMC2092432 17900354

[B12] Subhas N Patel PV Pannu HK Jacene HA Fishman EK Wahl RL Imaging of pelvic malignancies with in-line FDG PET-CT: case examples and common pitgalls of FDG PET RadioGraphics 2005 25 1031 1043 10.1148/rg.254045155 16009822

[B13] Hoh CK Hawkins RA Glaspy JA Dahlbom M Tse NY Hoffman EJ Schiepers C Choi Y Rege S Nitzsche E Cancer detection with whole-body PET using 2-[18F] fluoro-2-deosy-D-glucose J Comput Assist Tomogr 1993 17 582 589 10.1097/00004728-199307000-00012 8331230

[B14] Schiepers C Filmont JE CZernin J PET for staging of Hodgkin's disease and non-Hodgkin lymphoma Eur J Nucl Med Mol Imaging 2003 30 Suppl 1 S82 S88 12719922 10.1007/s00259-003-1165-6

[B15] Schoder H Larson SM Yeung HW PET/CT in oncology: integration into clinical management of lymphoma, melanoma, and gastrointestinal malignancies J Nucl Med 2004 45 Ssuppl 1 S72 S81 14736838

[B16] Nogami M Nakamoto Y Sakamoto S Fukushima K Okada T Saga T Higashi T Senda M Matsui T Sugimura K Diagnostic performance of CT, PET, side-by-side, and fused image interpretations for restaging of non-Hodgkin lymphoma Ann Nucl Med 2007 21 189 196 10.1007/s12149-007-0015-1 17581717

[B17] Specht L 2-[18F]fluoro-2-deoxyglucose positron-emission tomography in staging, response evaluation, and treatment planning of lymphomas Semin Radiat Oncol 2007 17 190 197 10.1016/j.semradonc.2007.02.005 17591566

[B18] Barrington SF O'Doherty MJ Limitations of PET for imaging lymphoma Eur J Nucl Med Mol Imaging 2003 30 Suppl 1 S117 S127 12748831 10.1007/s00259-003-1169-2

[B19] Mantzarides M Papathanassiou D Bonardel G Soret M Gontier E Foehrenbach H High-grade lymphoma of the bladder visualized on PET Clin Nucl Med 2005 30 478 480 10.1097/01.rlu.0000167482.23562.ab 15965322

